# Effect of environmental education on the knowledge of aquatic ecosystems and reconnection with nature in early childhood

**DOI:** 10.1371/journal.pone.0266776

**Published:** 2022-04-27

**Authors:** Maria João Feio, Ana Isabel Mantas, Sónia R. Q. Serra, Ana Raquel Calapez, Salomé F. P. Almeida, Manuela C. Sales, Mário Montenegro, Francisca Moreira

**Affiliations:** 1 Marine and Environmental Sciences Centre, Faculty of Sciences and Technology, University of Coimbra, Coimbra, Portugal; 2 Department of Biology, GeoBioTec–GeoBioSciences, GeoTechnologies and GeoEngineering Research Centre, University of Aveiro, Aveiro, Portugal; 3 CEIS20—Centre of Interdisciplinary Studies & MARIONET- Associação Cultural, Faculty of Arts and Humanities, University of Coimbra, Coimbra, Portugal; 4 MARIONET- Associação Cultural, Coimbra, Portugal; Universidade Regional Integrada do Alto Uruguai e das Missoes, BRAZIL

## Abstract

Blue and green ecosystems are considered a key for the improvement of cities sustainability, providing numerous ecosystem services and habitat for many species. However, urban streams are still neglected and degraded, specially in southern European countries. One important step towards the rehabilitation of these ecosystems is the awareness of their importance by citizens. This study aimed to assess the effect of 1-year of activities (field and laboratory) of an environmental education project on primary school children, in improving their knowledge on urban stream ecosystems and their problems. We analyzed students’ questionnaires before and after field and laboratory activities, drawings and group interviews. Initially, most children had incipient contact with rivers and streams, showing fears and lack of knowledge about them. As the project progressed, their perceptions changed, with a clear increase in the proportion of students recognizing the biodiversity associated to rivers (e.g., names of riparian trees, aquatic plants and invertebrates). Also, their fears decreased significantly, while their awareness to the impacts of artificialization and lack of riparian vegetation increased. Our results show that direct contact with nature have a positive role in the way it is understood by children, as well as promoting responsible and sustainable behaviors, being effective from the early primary-school years.

## Introduction

The process of urbanization witnessed since the second half of the 20th century has exerted a major impact on natural resources. In urban areas, the construction of roads and residential infrastructures has led to a progressive destruction of natural environments jeopardizing the sustainability of cities from an environmental, social, and economic point of view [1–3]. Particularly the aquatic ecosystems have been highly neglected, and many are polluted, artificialized, or fully covered as result of the increasing urbanization [4–8]. In consequence, the population often ignores their existence, their functions and services and contribute to their increasing degradation (e.g., [9, 10]). However, urban rivers and streams and their ecosystems, virtually present in all cities, offer blue (water) and green (riparian vegetation of the banks and channel) areas with a great potential to promote cities sustainability. If well preserved, their aquatic and terrestrial associated environments can support a wide biodiversity (e.g., birds, amphibians, reptiles, small mammals, fish, invertebrates, and aquatic plants), green corridors (through their riparian vegetation) among disconnected natural areas, and provide innumerous services to cities’ population [11]. Among these ecosystem services are: the improvement of cities resilience to climate changes, air, water and soil quality; providing a better city aesthetics; and areas to practice sports, for relaxation, or educational activities near schools [11].

People living in urban areas are affected by the environmental and social degradation being subject to daily constraints, obstacles, and pollution (e.g., noise or vehicle emissions, light pollution) with negative effects on their physical and mental health [12, 13]. The exposure to natural environments can compensate for these drawbacks, promoting human health and wellbeing through physical activity, stress reduction, social integration, and cooperative and environmentally sustainable behaviors (e.g., [14–17]). This beneficial and restorative effects associated to natural environments can be a result of conditioning and associative learning [18]. Thus, the early exposure of children to nature is fundamental for the creation of a positive experience, establishing the basis for sustainable behaviors [17, 19, 20]. However, in the cities children spend most of their time indoors and have little opportunity to learn in natural environments [21–25]. A survey carried out in the United Kingdom in 2009 shows that less than 10% of the children play in nature, while 40% of the adults said they did it when they were children [26]. In Portugal, as in many southern European countries, classes or school activities in natural environments are still rare as are the scientific experiments carried out during primary school years.

Building engagement with nature into school curricula has been proposed as a low-cost method to improve children’s psychological wellbeing [27, 28] and even academic attainment [29]. One way of achieving that is by bringing children to natural areas and implement hands-on activities. The opportunity to experiment facilitates the creation of bonds with the environment, and the community (e.g., [20, 23, 30]). Also, children must have the opportunity to act and contribute to transformation from an early age and actively participate in decision-making processes [31]. However, they are usually not included in discussions about problems, which limits their civic participation and their connection to the place [20, 32, 33].

In view of these, the environmental educational project CresceRio was created in 2018 in the city of Coimbra, Portugal, assuming the urgency to promote the preservation and restoration of urban streams, to reconnect the population of the city with nature, and the importance of children as present and future agents of transformation of societies ([34]; https://www.facebook.com/cresceriocoimbra/). During their primary school years, the same children participate in field and laboratory activities aiming to show them: 1) the unknown urban stream ecosystems near their schools and homes, their biodiversity and services; and 2) the problems of these streams resulting from anthropogenic pressures, and plan solutions through hands-on activities.

Here, we investigated the effect of this project on 6–7 years old children, aiming to assess if this could be a useful approach to be integrated in future educational programs in early primary school years. We expected an increased engagement with nature over time, scientific knowledge on freshwater ecosystems, and understanding of their problems associated to the urbanization process.

## Materials and methods

### Project actions

The project CresceRio was implemented in the primary schools of Eugénio de Castro, located in the center of the city of Coimbra (Central Portugal) by researchers of the University of Coimbra and the Marine and Environmental Sciences Centre (MARE), and two non-profit associations (the cultural association MARIONET and the environmental association PROAQUA). The city of Coimbra, with ca. 150.000 inhabitants, has temperate Atlantic climate and a hilly orography, being rich in stream catchments that flow into the main river that crosses the city, the Mondego River. Many of the streams are channelized and altered by centuries of urbanization (since pre-roman age), while others, in more recent urbanized districts are better preserved. Therefore, they have different ecological quality, biodiversity and provide different ecosystem services [11, 35, 36].

**Fig 1:**
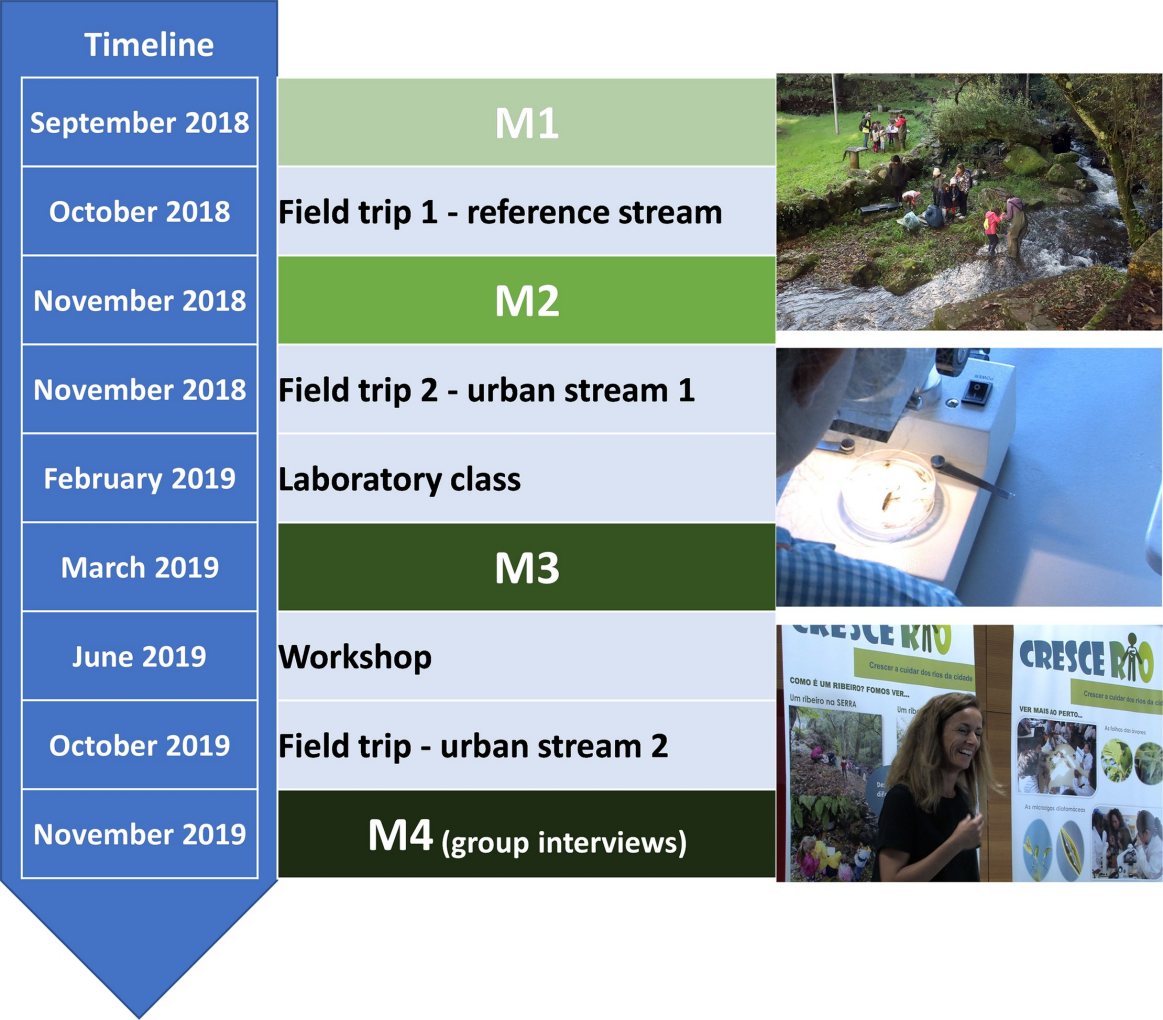
Timeline of CresceRio actions (field trips and laboratory class) and surveys to students (inquiries– M1-M3 and interviews—M4).

This study is focused on the activities undertaken with one class of 24 students over ca. 1 year. The hands-on activities included: 2 field trips and 1 laboratory class and 1 workshop undertaken in the 1st school year (2018–2019); and 1 field trip undertaken in the 2^nd^ school year (Fig 1). The progress of the students was assessed at 4 survey moments by questionnaires (3) and group interviews (1).

Fieldwork consisted in sampling two major elements of stream communities and bioindicators, the microalgae (diatoms) and benthic aquatic macroinvertebrates. In addition, the diversity of riparian vegetation, amphibians, terrestrial insects and birds and the presence of non-native and invasive species was also investigated *in situ* by the students, with the help of researchers, and registered. The most representative species were identified using simplified guides designed for children by the teams. Finally, the anthropogenic alterations in the channel and margins, such as cuts of vegetation, presence of weirs and dams, impervious surfaces, presence of litter, among others were also analyzed.

The first field trip (October 2018) was to a well-preserved reference stream located outside the city. This visit intended to provide the perception of a non-impacted stream ecosystem and its components (e.g., riparian gallery, diversity of aquatic habitats, flow conditions, substrate diversity, banks material, fauna and flora). The second field trip (November 2018) was to an urban stream near the school (ca. 1 km) with significant visible alterations. The stream is surrounded by buildings, roads and bridges, is linearized, has cuts in the riparian vegetation, presence of invasive species, and evidence of eutrophication. In a third activity (February 2019), samples of benthic invertebrates and diatoms were taken to the school and a laboratory environment was created in the classroom. The researchers guided the students through the processes of sorting and identification of macroinvertebrates and diatoms with stereo and binocular microscopes. In addition, they identified fallen leaves of the typical riparian trees collected by the students during the field trips. Then, the official biological quality indices used in Portugal for invertebrates and diatoms were calculated [37], the results discussed and compared to their observations in the field and photographs. In June 2019, end of the 1^st^ school year, the students participated in a workshop (that was also intended for other students that were not directly involved in the project) where they revisited their previous activities through photos and videos, saw again the benthic invertebrates and microalgae, and discussed the problems of urban streams. Finally, in the 2^nd^ school year the same students participated in a third field trip (October 2019) to a different urban stream, where they performed similar activities to those undertaken in previous ones (Fig 1). This second urban stream was less altered than the first, had a higher biodiversity, but still had visible signs of the impact of urbanization (crossed by a bridge and a road, stone walls in a small river stretch and cuts of riparian vegetation).

### Assessment of students’ perceptions and evolution

Questionnaires, interviews, and drawings were used at 4 survey moments (M; Fig 1) to assess the student’s evolution in terms of knowledge and awareness on urban freshwater ecosystems gained over the project. The questionnaires were made in a simple and direct language, in Portuguese (children’s native language) and employed common names of the species or large taxonomic groups, using the same terms that they heard in the field trips and laboratory classes. To simplify the term “river” was used throughout the questionnaires. Considering the difficulties that children could have in filling the questionnaires, given that they were carried out at the beginning of their literacy, we used symbols instead of writing (for example, smiling faces for yes and sad faces for no). Emotions are increasingly used as a communication system recognized by both children and adults [38]. Also, the teacher in the classroom administered the questionnaires but was not allowed to explain the meaning of the terms used in the questionnaires. The first questionnaire was conducted before the first field trip (September 2018, M1); the second, after the first field trip (November 2018, M2) and the third after the second field trip and the laboratory class (March 2019, M3) (Fig 1). In M1, 23 children (14 girls; 9 boys) with 5–6 years old were surveyed; in M2, 22 children (13 girls; 9 boys) and in M3, 24 (14 girls; 10 boys) with 6–7 years old.

The questionnaire was composed of five groups of questions (Q): 1) **students’ identification and background:** student number–Q1, age–Q3, gender—Q4; where the student live—municipality–Q2 and country or city—Q5; 2) **awareness of streams and rivers:** if there is any river/stream near their homes–Q6 and its name–Q7; if they know any a river–Q8 and where it is located–Q9; if they visit rivers/streams- Q10 and with whom—Q11 (4 options) and when they go–Q12 (4 options); and what they do in the rivers/streams–Q13 (8 options); if they think it is dangerous to go to a river/stream—Q14, and what could constitute a risk–Q15 (4 options); 3) **recognition of the biodiversity associated to rivers:** if there are animals in the rivers–Q16 and which ones—Q17 (11 options corresponding to large groups, e.g., fish, mosquitos, dragonfly, amphibians); if there are plants inside the rivers (aquatic plants)–Q18 and which ones (3 options)–Q19, if there should be trees in the river banks (riparian vegetation)–Q20, and which ones (5 options—common names of tree species)–Q21; 4) **awareness of stressors and alterations affecting rivers:** what is wrong in a river/what should not be present—Q22 (29 options that included natural features, such as mud, sand, stones or boulders and also indicators of impairment such as colors in the water, absence of curves, presence of litter, construction in the banks and margins, artificialization of the channel and banks); and 5) **awareness of the ecosystem services provided by rivers to the population**–Q23 (11 options). In most of options the students needed to choose the categories “Yes” or “No” and in some cases any of those (no selection). All questions are listed in S1 Table.

As a part of the questionnaires at M2 and M3, children were asked to draw what they expected to find in a river, given that at this age they are more used to drawing than to writing and would give them more freedom to represent what they learned and remember. The elements represented in the drawings were listed and analyzed.

In addition, in November 2019 (2nd of the project) students were interviewed in groups (M4; Fig 1). By this time the students had turned 7 years old. The group interviews allowed to: 1) test the consistency of the responses to the questionnaires; 2) evaluate the result of the activities carried out up to that time and not included in the interviews; 3) to further investigate some questions, namely their awareness of river conservation and protection actions, as these aspects were discussed with them during the last activities. The interviews were carried out in four groups (G) of students composed of five children and one by four, for a total of 24 students. All groups included boys and girls. The focus groups lasted 30 minutes and took place in a room provided for this purpose at the school. The interviews focused on the questions of the survey, which were deepened. Students were left free to speak and introduce new topics. All interviews were tape recorded and transcribed verbatim.

### Data analyses

The characterization of the target group—students and their habits (e.g., where they live, how they travel to the school, if they visit streams and rivers or not) resulted from the joint analysis of the results of all questionnaires for these questions and interviews. The questions with their various options were used as variables (except for gender and home location). To evaluate the significance (p<0.05) of differences in the recognition of the biodiversity associated to rivers, paired t-tests were applied to the answers of the first questionnaire moment (M1) and the last questionnaire moment (M3). The results between the three questionnaires (M1, M2 and M3) were compared through graphical analyses, a Multiple Correspondence Analyses (MCA) and associated Chi-Square tests.

To assess the existence of a global temporal pattern we used the Multiple Correspondence Analysis (MCA) based on the answers of the students to the categorical variables in questions Q6—Q23. Moment (M) and Gender were treated as supplementary variables (thus not contributing to the spatial patterns). An absence of reply was treated as missing value. The information from one student in M2 was eliminated due to the high number of missing values. The missing values were not replaced by simulated values because they may be an indicator of hesitation and lack of confidence. The correlation coefficients (R^2^) of the supplementary variables Moment and Gender with the MCA first dimensions were used to assess the importance of these in explaining the students’ answers. Finally, Chi-square tests were used to test if the categorical variables have significant variation (p<0.05) over time (M). The more significant the test is, the more Ms and answers are linked.

For graphical and statistical outputs, we have used Microsoft Office 365 and R software [39] using FactoMineR [40], and ggplot2 [41] packages. The treatment of the interviews and their consequent systematization was done based on the technique of content analysis, which aims to simplify and organize the raw data.

### Ethics statement

This study was approved by the board of the school to which the students belonged too (Agrupamento de Escolas Eugénio de Castro). The parents or tutors of the children involved in this study gave their written consent to the participation of students in the study. This study was fully performed in the presence of the responsible teacher and in the context of the programmatic content of primary school years, and following the rules a priori established by the school board and teacher (for field, laboratory and surveys). The data was analysed anonymously (S1 Data).

## Results

### Students’ background

According to the results of the questionnaires 83% of the students live in urban areas. The interviews with the focus groups revealed that most children travel to school by car (14). They are more used to play indoors, although they find more attractive to play outside their homes to have more freedom. The different answers provided were:

- There are more things at home, at home we have our toys; but on the street we can climb trees, we can play
- On the street, because we can investigate things and we can see new things that we’ve never seen before

### Awareness of streams and rivers

In M1, when the project began, most children (78%) said that there is no river close to home, but this number decreased over time to 50 and 54% in M2 and M3, respectively. There was also an increase in the number of children who claim to know a river (from 56% in M1 to 82 and 83% in M2 and M3, respectively). Yet, most children say “They usually go to the river” (61% in M1, 68% in M2 and 58% in M3) and this activity is done with family and friends. Almost all activities that can be done in the river (e.g., walk the dog, picnic, swimming) are mentioned by the students although the most frequent one is family outings (between 39% in M1, 50% in M2 and 54% in M3). Over time, more students started to report more activities at the river (from 24 activities chosen by the class in M1 to 59 in M3). In the interviews, when asked about what they do in the river, children showed a great attraction for water by answering:

- I put my hand in the water, almost the whole arm
- I swim and dive
- I go to the river beach on the Mondego River, dive, (…)
- I like to put my feet in the water
- I like to put my hand on the river and play by the river and see the little fish
- To set foot in the water

**Fig 2:**
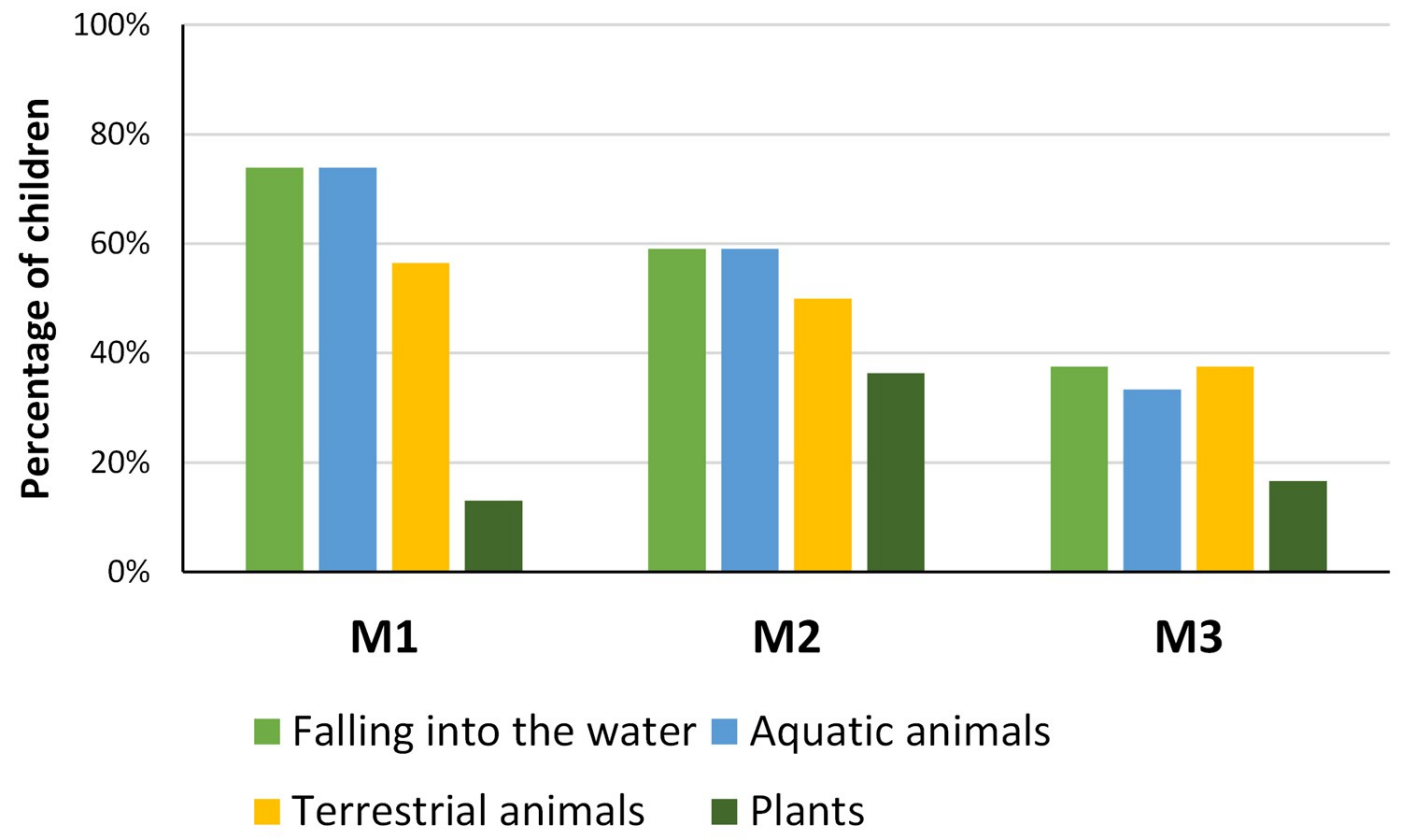
Percentage of children referring dangers in the rivers/streams (falling into the water, aquatic animals, terrestrial animals, and plants) over time in the questionnaires (M1-M3).

Although these numbers are not consistent over time, most students (70% in M1, 46% in M2 and 83% in M3) think that rivers are not dangerous. But when questioned about what can be dangerous in a river, they point out more frequently falling into the water and the aquatic animals, followed by terrestrial animals (Fig 2). Individually, the importance of these dangers decreases over time in a consistent way, except for the risk by plants that is always low.

The interviews support these conclusions, but it should be noted that some animals that they are afraid of, are not actually found in Portuguese rivers, such as sharks, or alligators, animals linked to their imaginary. To the question “Are you afraid of anything?” they answered:

- Lesser weever
- Piranhas
- Fishes, sometimes.
- I’m afraid of… I’m afraid of very big fishes
- I’m afraid of eels, (…)
- Fishes
- Bees
- Sharks and eels

### Recognition of river biodiversity

**Fig 3:**
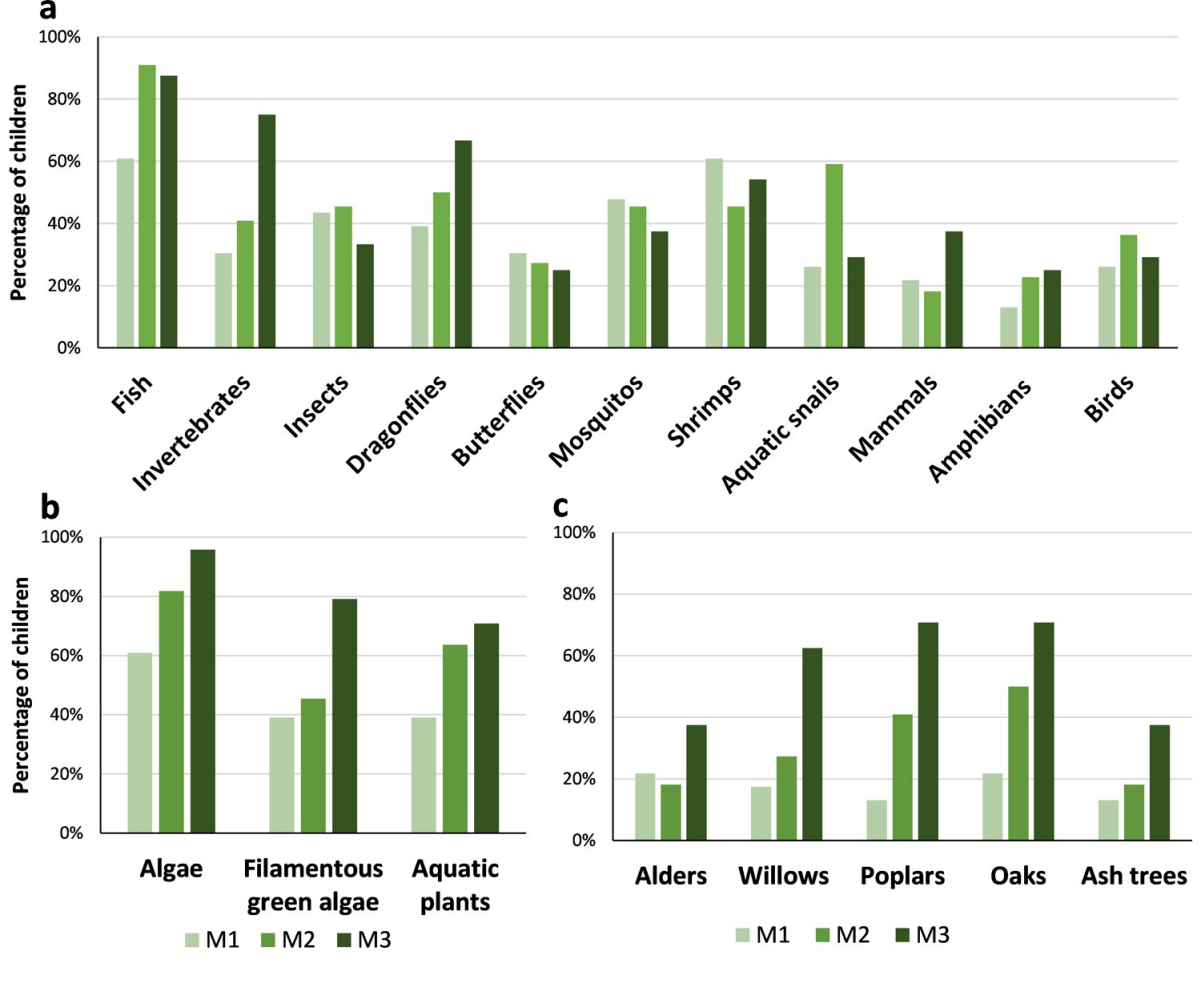
Percentage of students recognizing the existence of elements of the fauna and flora of stream ecosystems over time (M1-M3) in the questionnaires.

Between M1 and M3 there was an increase in the proportion of students recognizing the existence of animals, aquatic plants, and trees near or in the river: 57% in M1, 86% in M2 and 96% in M3. The most notorious increase of awareness was in aquatic invertebrates, followed by dragonflies but there were also increases in amphibians and birds (Fig 3A). The same also applies to aquatic plants (algae, filamentous algae and aquatic plants/macrophytes; Fig 3B): 39% in M1, 68% in M2 and 92% in M3. And for riparian vegetation, the recognition that they are part of the riverine ecosystems increased from 35% in M1 to 46% in M2 and 88% in M3, with a similar tendency for all categories (alders, willows, poplars, oaks and ash trees; Fig 3C).

When asked during the interview whether there should be trees by the river and why, most children answered affirmatively, emphasizing its importance for the survival of animals and humans by replying:

- Because invertebrates can eat leaves
- When they fall
- For us
- They give us oxygen.

In addition, the students recognize the importance of not cutting the riparian vegetation, when asked if they could be cut by answering:

- No, because otherwise we don’t have oxygen and we die
- And the reeds appear and destroy the rivers

**Fig 4:**
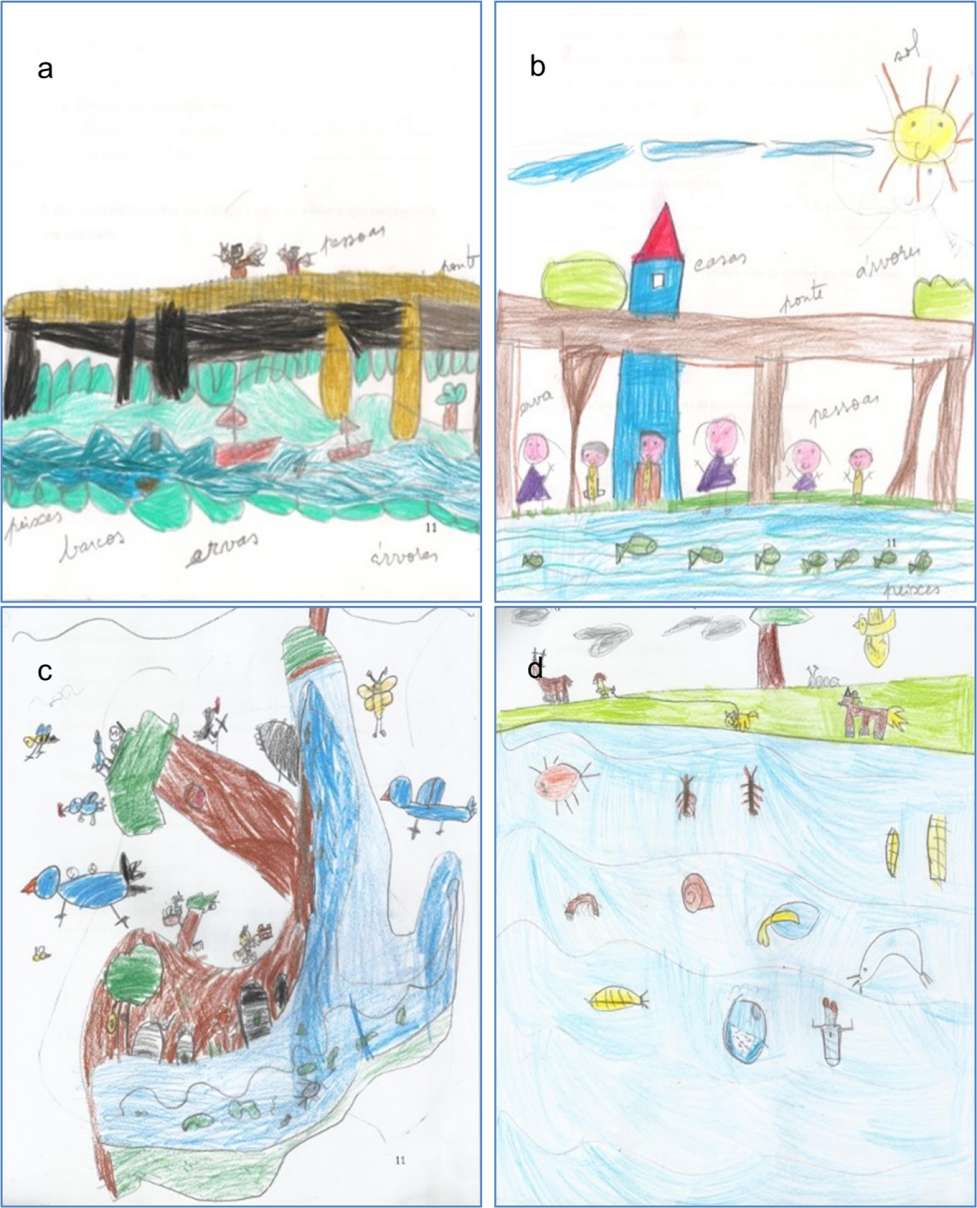
Examples of drawings made by children in the M2 –after the first field trip (a, b) and M3 –after the second field trip and laboratory class (c, d).

**Fig 5:**
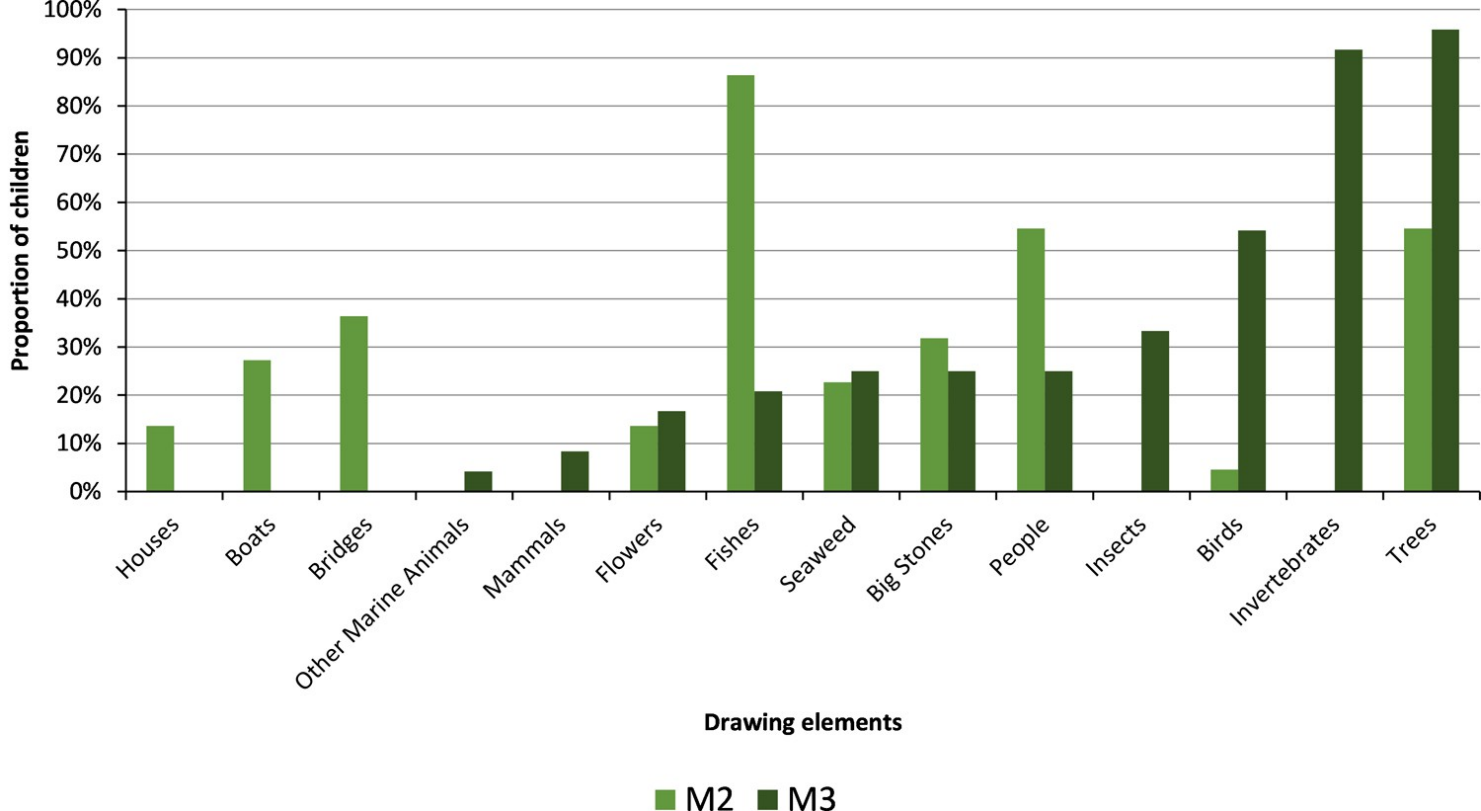
Proportion of students representing different elements related to alterations and biodiversity in their drawings made along with questionnaires of M2 and M3 moments.

The analysis of the drawings (Fig 4) made by the students also showed that there was a clear change in the perception of riverine biota over time. One of the greatest differences was the fact that the imaginary and the common ideas of what a river is, gave way to the reality observed during the field trips and laboratory class. Between M2 and M3, the main change observed is related to the introduction of invertebrates, which are represented in detail in most drawings of M3 (92%). Also, the proportion of drawings in which trees and birds are represented also increased considerably (Fig 5). Insects, birds and mammals that did not come out in the first drawings appeared also in the second ones. On the other hand, the proportion of drawings depicting fishes and people decreased. In turn, elements such as bridges, houses and boats are no longer represented in M3.

### Awareness of stressors and alterations affecting rivers

**Fig 6:**
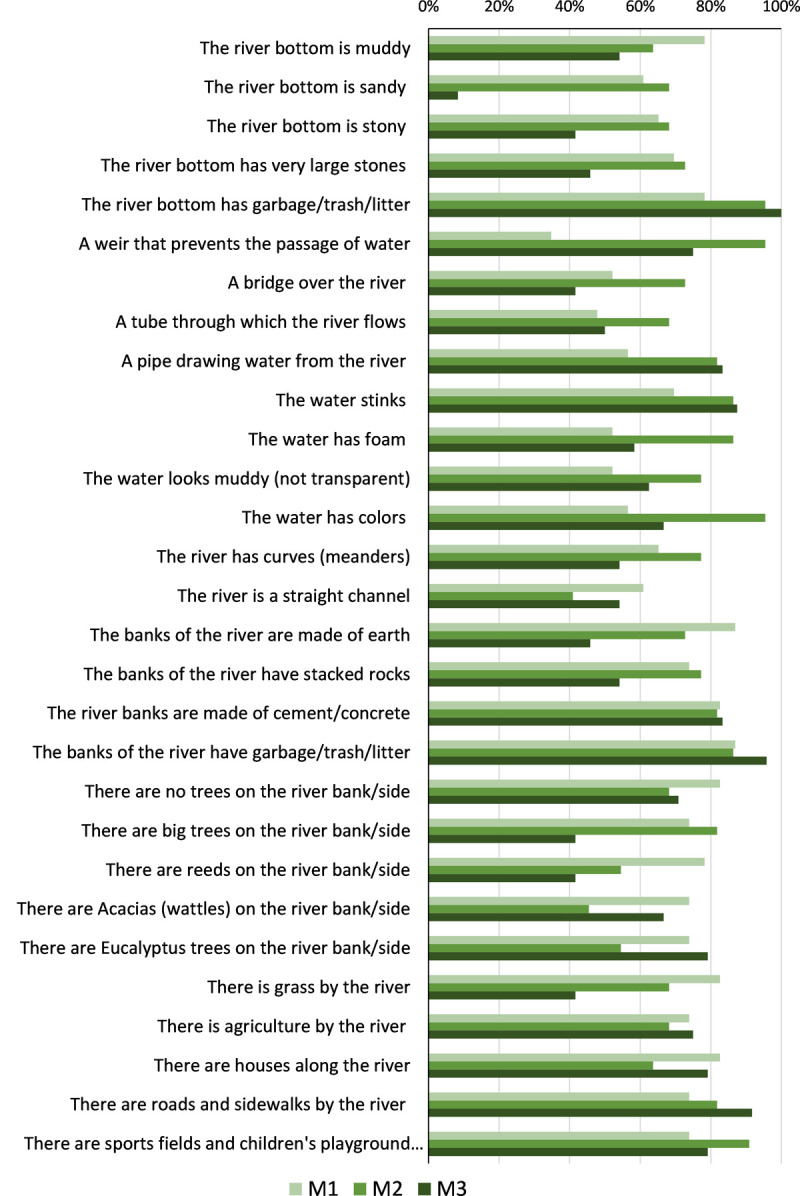
Proportion of students mentioning what they perceive as wrong in a river (or that shouldn’t be there) in M1, M2 and M3.

The students revealed some difficulties in distinguishing stressors and alterations of rivers (what shouldn’t be there) against natural features (that could be there). Yet, when looking into individual items, there was also a clear evolution over time, specially between M1 and M2 (Fig 6). This is more evident regarding the natural substrates in the channel (e.g., stones and sand) or earth covering the river banks, where the proportion of responses saying they shouldn’t be there decreased. And in the presence of some artificial elements (e.g., presence of water abstraction, grass in the river banks, roads and sidewalks, bad smell, garbage), where there was an increase in the proportion of answers saying that they should not be present in a river. Also, the *Eucalyptus* trees (exotic), *Acacia* (exotic and invasive trees), houses and agriculture are perceived as wrong aspects in river banks in a similar way in M1 and M3.

In the interviews, the litter in the river is very present in the students’ discourse as one of their main problems:

- From the dirty river, with plastics and other water bottles.
- Plastic on the floor.
- I like plastics less.
- The garbage and trash.
- Trash and garbage.

When asked about what is wrong in the rivers, in addition to litter and trash, students also mention the reeds, buildings, cement and dirty water and absence of animals or trees by replying:

- No trees around it
- No animals
- Without stones
- And also without animal food
- Dirt
- The reeds
- Dirty water
- Large stones
- The stones are not bad because the animals sometimes live there
- The reeds are very bad

### Awareness of river ecosystem services

**Fig 7:**
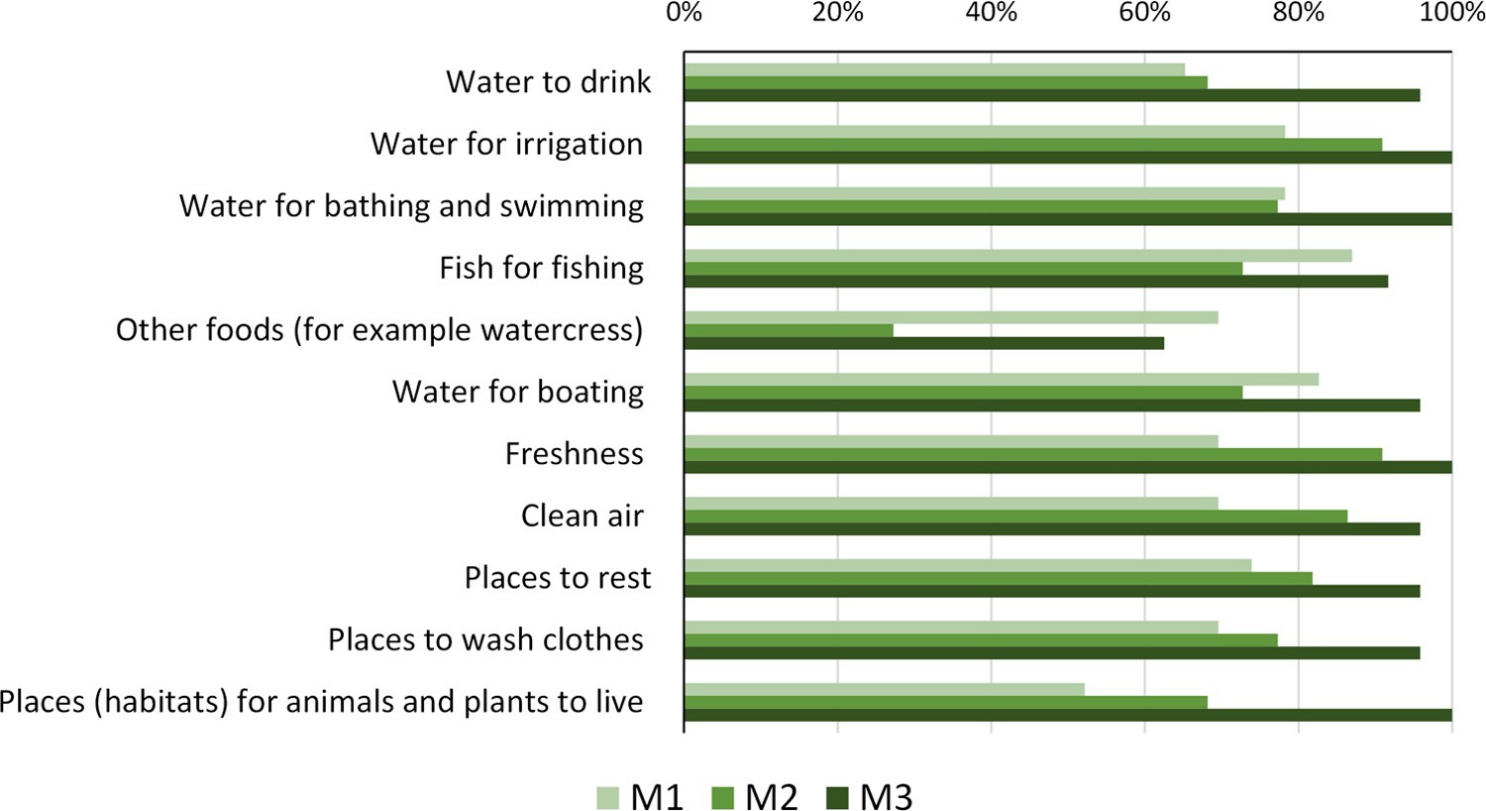
Proportion of students mentioning what they perceive that a river or stream could give them (ecosystem services) at M1, M2, M3.

From M1 to M3, children became aware of the services that could be provided by a river or a stream (Fig 7). In the M3 it is clear to all children that the river offers water for irrigation, a place to swim and bath, freshness, and that it provides habitat for animals and plants.

### Global effect of the project activities in the awareness and knowledge of river ecosystems over time

**Fig 8:**
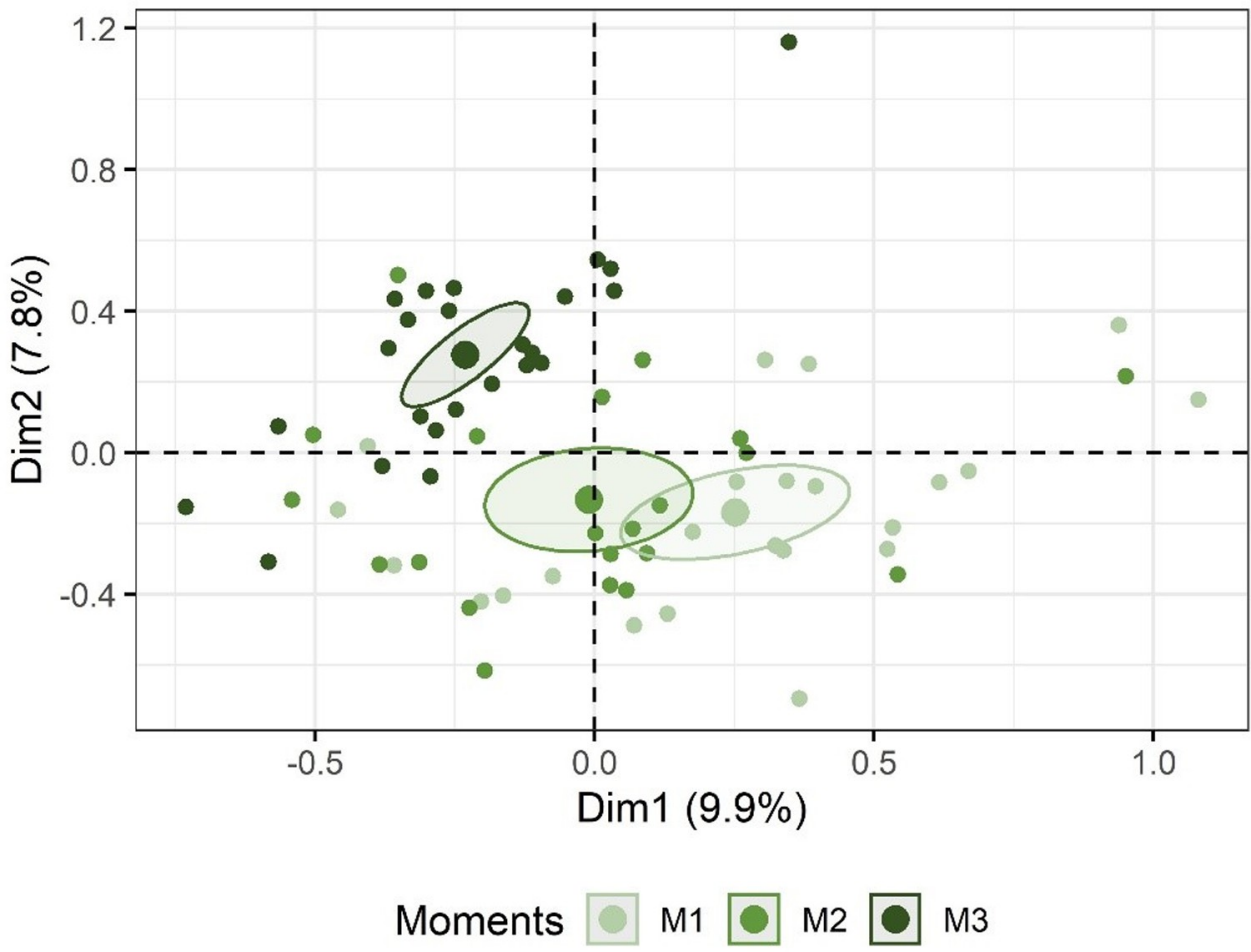
Projection of individuals (Child+M) obtained in a multiple correspondence analysis (MCA) performed on categorical variables. Confident ellipses for Moments were set at 95% confidence level.

In the Multiple Correspondence Analysis (MCA) (Fig 8) the first two dimensions of MCA explained 18% of data variability (Dim1 = 10% and Dim2 = 8%). Over these two axes, the answers show a clear temporal gradient, from M1 to M3, with a smaller segregation between M1 and M2 and a larger segregation between M3 from M2 (also over Dim2), which is confirmed by the significant correlations of M with Dim1 (R^2^ = 0.28, p< 0.001) and Dim2 (R^2^ = 0.37, p< 0.001). Gender presented low correlation coefficients with both Dim1 and Dim2 (R^2^ = 0.09, p = 0.04), and thus had a small contribution to the segregation of M.

The Chi-square tests (Table 1) reinforce the results of the above sections, showing a high number of variables correlated with both dimensions (along which M is correlated). The most significant correlations (p<0.001) highlight that the students became more aware of rivers they visit, and of the presence of aquatic plants and riparian trees, of the river names. In addition, they modified their perceptions towards the natural (e.g., sandy bottom, earth in the margins) and artificial elements of the streams (as weirs), and the services provided by these ecosystems (e.g., freshness, habitat for animals and plants).

**Table 1:** Results of significant p-values (p<0.05) for the Chi-square test between the Moments (M) and the categorical variables.

Variables	p-value	df
Q9. Where is the river located?	0.001	8
Q12.3. (Go to a river) on weekends	0.019	2
Q12.4. (Go to a river) on holidays	0.007	2
Q14. Believe rivers are dangerous	0.014	4
Q16. (There are) animals living in the river	0.015	4
Q17.2. (There are) Invertebrates (in the river)	0.012	4
Q18. (There are) plants inside the river	0.002	4
Q19.1 (There are) algae in the river	0.012	2
Q19.2. (There are) filamentous algae in the river	0.020	4
Q20. Are there trees on the river bank?	0.002	4
Q21.2. Willows live by the river	<0.001	4
Q21.3. Poplars live by the river	<0.001	4
Q21.5. Ash trees live by the river	<0.001	4
Q22.2. (There shouldn’t’ be) sand in the river channel	<0.001	2
Q22.5. (There shouldn’t’ be) trash in the river	0.005	2
Q22.6. (There shouldn’t’ be) a weir	<0.001	4
Q22.9. (There shouldn’t be) a pipe drawing water	0.042	2
Q22.11. (There shouldn’t’ be) foam in the water	0.048	2
Q22.13. (There shouldn’t’ be) colors in the water	0.013	2
Q22.16. Banks made of earth	0.007	2
Q22.21. Big trees on the river bank	0.005	2
Q22.25. Grass by the river	0.010	2
Q23.1. Water to drink	0.021	2
Q23.2. Water for irrigation	0.049	2
Q23.3. Water for bathing and swimming	0.040	2
Q23.5. Other food (e.g., watercress)	0.005	2
Q23.7. Freshness	0.007	2
Q23.8. Clean air	0.048	2
Q23.11. Habitats	0.001	2

### Awareness of river conservation and protection actions

Finally, during the interviews, when questioned about what should be done to preserve the streams and rivers, the students referred the issue of garbage but also the deforestation, the growth of (invasive) reeds (*Arundo donax* is a very common invasive species in the banks of rivers in the region), the recovery of streams’ morphology and also the communication with adults:

- Do not throw trash on the floor
- We must not straighten the rivers.
- Don’t put big stones
- Cement
- Not to cut down the trees
- Not to cut the leaves because the animals eat them
- Take out the reeds
- Remove the big stones
- Put more animals.
- Clean up the trash
- Take out the bottles
- Take out the reeds
- Do not plant things there that are not from that place and do not throw trash on the ground, do not put more reeds because it is difficult to remove.
- They grow a lot and it’s hard to pull them out, we have to burn them
- (Children) should tell all adults not to make the rivers dirty, not to do anything that disturbs the rivers.
- And also when we are adults we must not forget
- I’m always telling my mom things I learn at school, not to do those things

## Discussion

This study revealed three main findings: 1) children of primary schools located in an urban environment had a poor contact with rivers and streams, and nature in general; 2) after 1 year of activities and close contact with streams, their knowledge on the aquatic and terrestrial biodiversity associated to rivers, and the awareness of their main problems increased significantly while, their fears and concerns decreased; 3) the continuation of the project over time is a key for its success, as marked differences in students’ behavior and knowledge were only clear after three activities and some months of project and not immediately after the first field trip.

Throughout this study, we found that most children involved in the project had sporadic contact with the rivers and streams, although they live in its proximity. They visit rivers mainly during summer holidays and weekends, mostly for family outings and picnics. The general poor contact with nature may explain the fears that the children mention and those that they showed during field visits to the streams, especially in the beginning of the project. Several children were afraid of falling into the water, of animals and getting dirty. Similarly, Mahidin and Maulan [20], concluded that children, despite recognizing the beauty of nature, are afraid to contact with it. This may be justified by the absence of learning about the functions and benefits of nature. Their incipient contact with nature leads, in some cases, to the development of unreal ideas that are present in the fears and concerns identified by some students. But although they are afraid to interact with the “animals of the river” and with the “animals of herbs”, they are fascinated by them. In fact, what attracts them most in the streams are animals and water, elements that appear mentioned in several studies [42–44]. However, over time their fears have consistently decreased. This supports the idea that direct contact with the environment will facilitate the deconstruction of fears associated with the natural environment [20, 23, 45].

The lack of knowledge that the students showed about the animals that inhabit the streams and the trees that surround them, also demonstrates how far they are from nature. In the case of trees, this distance is notorious, since they showed great difficulty in identifying very common species, such as oaks or willows. This does not mean, however, that they are not interested in their exploitation. Children in general, show a fascination and a natural appetite for natural environments that should be explored [46]. Also, Freeman and van Heezik [47], argued that although most children have today less contact with nature than those of previous generations, they are interested in the natural world, valuing it. Children are only temporarily disconnected due to factors such as the environment in which they live, the influences and pressures of peers and the assimilation of the ideas transmitted by the family. Indeed, according to several studies, culture seems to act on how individuals connect with nature [48, 49]. The interest shown by students in exploring the streams ecosystem during field visits and in the microscopic observations is evidence of this finding.

This interest for the activities proposed and the direct contact with the fauna and flora produced clear results in terms of their knowledge regarding aquatic invertebrates and riparian trees. Regarding invertebrates, as well as microalgae, the fact that they observed them in the classroom under the microscope can be associated with the easiness with which they recognize their presence in the streams and reproduce them in the drawings, as this was clearer again in M3 in opposition to M2. Additionally, trees were particularly represented in children’s drawings of the last questionnaire (M3), contrary to the initial ones, after the first field trip (M2). This increasing importance of trees may result from the contrast between the naturalized stream and the urban stream observed in the second field trip. In addition, in the laboratory class (before M3) they analyzed diverse leaves and images of riparian trees and learned their names, reinforcing the field observations. The importance of hands-on and outdoor activities, and systematic teaching in the scientific knowledge is well studied (e.g., [28, 29, 50, 51]. Our study also confirmed that environmental programs should not be restricted to sporadic activities as a longest duration is more likely to change behaviors [52].

Despite the physical relationship they establish with nature being more and more superficial, children are increasingly aware of environmental threats [21] and that was evident in group interviews (M4). The dangers that garbage and pollution pose to the environment and to animals, are often pointed out by students as a problem, showing themselves concerned with the actions of adults. Yet, the environmental perception that they showed is acquired second-hand, being associated mainly with the education programs and information conveyed by the media. Thus, there is a difficulty in identifying the specific problems of rivers and streams since they are less widespread. But although there are still some hesitations, there was an increased perception of the stressors and alterations to which rivers and streams are subject. The threat that the artificial elements constitute (e.g., weirs, dams, artificial walls, absence of trees, color or smell of the water) is highlighted by the students who showed concern for the valorization of the natural environment in detriment of the environment modified by the human being. Nevertheless, our results showed that this is a topic where more investment is needed, as not all concepts were clearly understood.

The development of environmental awareness is primarily concerned with the development of love for nature [46] given that “you do not love and respect what you do not know” (Louv, 2010). In fact, the project permitted students to interact *in loco* with the streams, which is helping to change some misconceptions about them. The fact that they experienced nature first hand, allowed them to fully understand it through all the senses (odors, sounds, textures), establishing a stronger connection with it. And the children started to understand more clearly the services that rivers and streams can provide (e.g., freshness, clean air, water for several purposes and some food items). The classroom research activities also played a major role in developing the scientific curiosity, completing field trips. Together all these activities led children to raise the value they give to nature, which constitutes a good promise for more sustainable cities. Despite the clear effect that project activities had on city children (the target of our project), these outcomes cannot be generalized to all children of this age, as others living in more rural areas would probably perceive nature in different ways. Thus, the gains of the project could also be different, likely more associated to the increase in scientific knowledge but less regarding the contact with nature.Finally, in this study we couldn’t detect differences between genders, including the fears related to rivers. Yet, further investigation should be done on this topic, as this was not the main aim of our questionnaires and it is still a fact that science studies and careers are globally uneven, with a lower proportion of girls pursuing them [53, 54].

## Conclusions

Although the conclusions are limited to the group under study, we can say that the continuity of environmental education activities of project CresceRio with the same group of students allowed for a change of perception about rivers and stream ecosystems and contributed to the acquisition of tools to develop critical and informed thinking. The school environment provided a real and effective opportunity to promote proximity to nature, in a consistent and over a long period of time [27, 28]. Our results are aligned with current outdoor learning movements that supports the mission of reconnecting children with nature, through ‘wild time’ and outdoor play and learning activities. Outdoors activities and biodiversity-focused programs bring physical benefits, such as preventing sedentary lifestyle and promoting mental and behavioral health while increasing children cooperation, academic performance and sustainable behaviors [28, 29, 50, 51]. And raising awareness in children on the importance of preserving and recover nature within cities and freshwater ecosystems is essential to promote their rehabilitation and create more sustainable cities.

## Supporting information

S1 Table List of questions made in the three questionnaires (M1, M2, M3) to the students and used as variables in data analyses. (DOCX)

S1 Data (XLSX)
