# Supplementary material for: Effect of environmental education on the knowledge of aquatic ecosystems and reconnection with nature in early childhood
Source: PLoS One. 2022 Apr 27;17(4):e0266776. doi: 10.1371/journal.pone.0266776 (PMC9045661; doi:10.1371/journal.pone.0266776)
Supplement: S1 Table — (DOCX) [file pone.0266776.s001.docx]

**Supporting Information** **Table 1 – List of questions made in the three** questionnaires (M1, M2, M3) to the students and used as variables in data analyses.

| **Groups of questions** | **Questions/Variables** | |  |
| --- | --- | --- | --- |
| **Student identification and background** | Q1 | Student number (1-24) | |
|  | Q2 | Municipality (nominal) | |
|  | Q3 | Age (5-7) | |
|  | Q4 | Gender (Girl/Boy) | |
|  | Q5 | Where do you live? (City/Countryside) | |
| **Awareness of streams and rivers** | Q6 | Is there a river near your house? (Yes/No) | |
|  | Q7 | What is the name of the river near your house? (nominal) | |
|  | Q8 | Do you know any river? (Yes/No) | |
|  | Q9 | Where is the river you know located? (Coimbra/Outside) | |
|  | Q10 | Do you go to any river? (Yes/No) | |
|  | Q11 | With whom do you go to a river? (Yes or No or No reply in the answers below) | |
|  | Q11.1 | Parents | |
|  | Q11.2 | Grandparents | |
|  | Q11.3 | Friends | |
|  | Q11.4 | School or extra-curricular activities | |
|  | Q12 | When are you going to the river? (Yes or No or No reply in the answers below) | |
|  | Q12.2 | During the week | |
|  | Q12.3 | On weekends | |
|  | Q12.4 | On holidays | |
|  | Q12.5 | During school time | |
|  | Q13 | What you do in the river? (Yes or No in the options below) | |
|  | Q13.1 | Walk with the family | |
|  | Q13.2 | Walk the dog | |
|  | Q13.3 | Picnic | |
|  | Q13.4 | Bathing/Swimming | |
|  | Q13.5 | Boating | |
|  | Q13.6 | Catching /throwing stones | |
|  | Q13.7 | Fishing | |
|  | Q13.8 | Wash clothes | |
|  | Q14 | Are the rivers dangerous? (Yes/No) | |
|  | Q15 | What is dangerous in the river? (Yes or No or No reply in the answers below) | |
|  | Q15.1 | Falling into the water | |
|  | Q15.2 | Aquatic animals | |
|  | Q15.3 | Terrestrial animals | |
|  | Q15.4 | Plants | |
| **Recognition of the biodiversity associated to river** | Q16 | Are there animals living in the river? (Yes/No) | |
|  | Q17 | Which animals live in the river? (Yes or No or No reply in the answers below) | |
|  | Q17.1 | Fish | |
|  | Q17.2 | Invertebrates | |
|  | Q17.3 | Insects | |
|  | Q17.4 | Dragonflies | |
|  | Q17.5 | Butterflies | |
|  | Q17.6 | Mosquitos | |
|  | Q17.7 | Shrimps | |
|  | Q17.8 | Aquatic snails | |
|  | Q17.9 | Mammals | |
|  | Q17.10 | Amphibians | |
|  | Q17.11 | Birds | |
|  | Q18 | Are there plants inside rivers? | |
|  | Q19 | What plants are within the river? | |
|  | Q19.1 | Algae | |
|  | Q19.2 | Threads of green algae (filamentous algae) | |
|  | Q19.3 | Aquatic plants (plants in the water) | |
|  | Q20 | Do you think there must be trees on the river bank? (Yes/No) | |
|  | Q21 | Which trees live by the river? (Yes or No or No reply in the answers below) | |
|  | Q21.1 | Alders | |
|  | Q21.2 | Willows | |
|  | Q21.3 | Poplars | |
|  | Q21.4 | Oaks | |
|  | Q21.5 | Ash trees | |
| **Awareness of stressors and alterations affecting rivers** | Q22 | What shouldn't be in a river? (Yes or No or No reply in the answers below) | |
|  | Q22.1 | Mud | |
|  | Q22.2 | Sand | |
|  | Q22.3 | Stones | |
|  | Q22.4 | Large stones | |
|  | Q22.5 | Garbage/trash/litter in the water | |
|  | Q22.6 | A weir that prevents the passage of water | |
|  | Q22.7 | A bridge over the river | |
|  | Q22.8 | A tube through which the river flows | |
|  | Q22.9 | A pipe drawing water from the river | |
|  | Q22.10 | Bad smell in the water | |
|  | Q22.11 | Foam in the water | |
|  | Q22.12 | The water looks muddy | |
|  | Q22.13 | Colours in the water | |
|  | Q22.14 | Curves (meanders) | |
|  | Q22.15 | A straight channel | |
|  | Q22.16 | Earth in the river bank | |
|  | Q22.17 | Stacked rocks in the river bank | |
|  | Q22.18 | The river bank are made of cement/concrete | |
|  | Q22.19 | Garbage/trash/litter in the river bank | |
|  | Q22.20 | No trees on the river bank | |
|  | Q22.21 | Big trees on the river bank | |
|  | Q22.22 | Reeds on the river bank | |
|  | Q22.23 | Acacias on the river bank | |
|  | Q22.24 | Eucalyptus trees on the river bank | |
|  | Q22.25 | Grass by the river | |
|  | Q22.26 | Agriculture by the river | |
|  | Q22.27 | Houses along the river | |
|  | Q22.28 | Roads and sidewalks by the river | |
|  | Q22.29 | Sports fields and children's playground by the river | |
| **Identification of the ecosystem services provided by streams/rivers to the population** | Q23 | What gives you the river? | |
|  | Q23.1 | Water to drink | |
|  | Q23.2 | Water for irrigation | |
|  | Q23.3 | Water for bathing and swimming | |
|  | Q23.4 | Fish | |
|  | Q23.5 | Other food (e.g., watercress) | |
|  | Q23.6 | Water for boating | |
|  | Q23.7 | Freshness | |
|  | Q23.8 | Clean air | |
|  | Q23.9 | Places to rest | |
|  | Q23.10 | Places to wash clothes | |
|  | Q23.11 | Habitat for animals and plants | |
